# Supplementary figures and images for: A docked mutation phenocopies dumpy oblique alleles via altered vesicle trafficking
Source: PeerJ. 2021 Oct 13;9:e12175. doi: 10.7717/peerj.12175 (PMC8520396; doi:10.7717/peerj.12175)

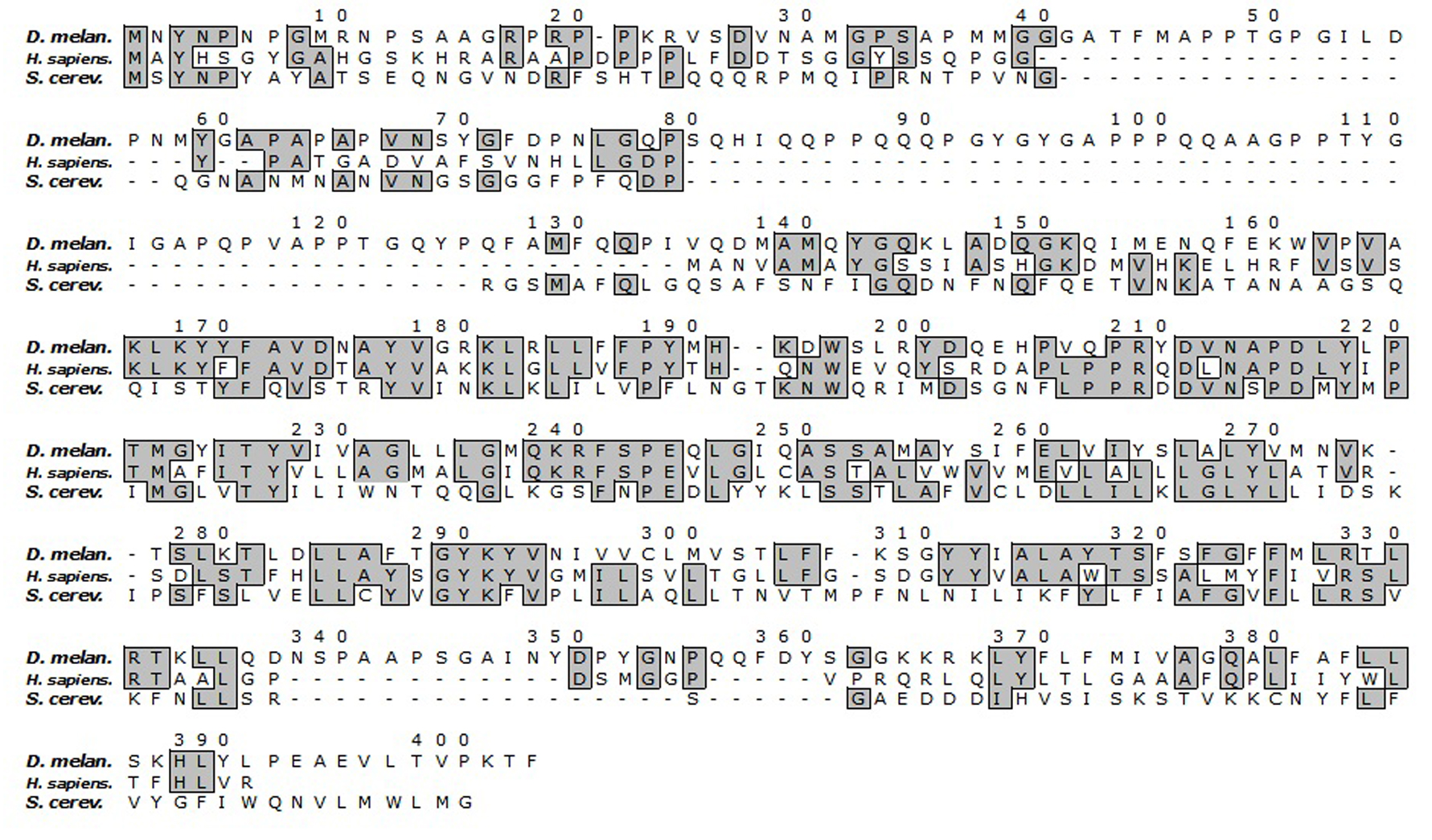

Supplement: Supplemental Information 1 — Alignment was generated initially by using the CLUSTAL implementation within the MacVector package, and then manually improved by eye. Residues identical among two or three of the sequences are shaded in dark gray. [file peerj-09-12175-s001.jpg]

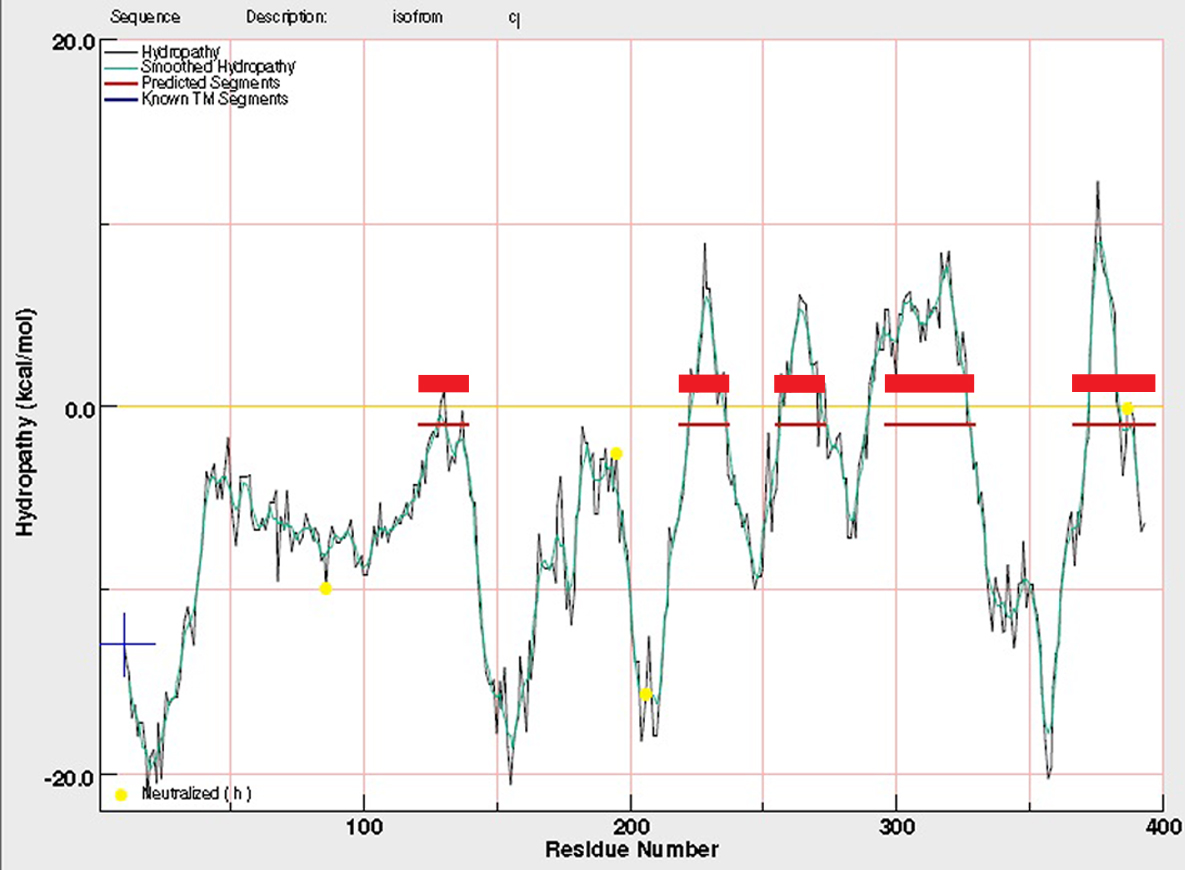

Supplement: Supplemental Information 2 — Plot was generated from the online MPEx tool available at https://blanco.biomol.uci.edu/mpex/(Snider et al., 2009). The position of five predicted transmembrane regions typical of YIF1 family members are indicated by red, horizontal rectangles. [file peerj-09-12175-s002.jpg]

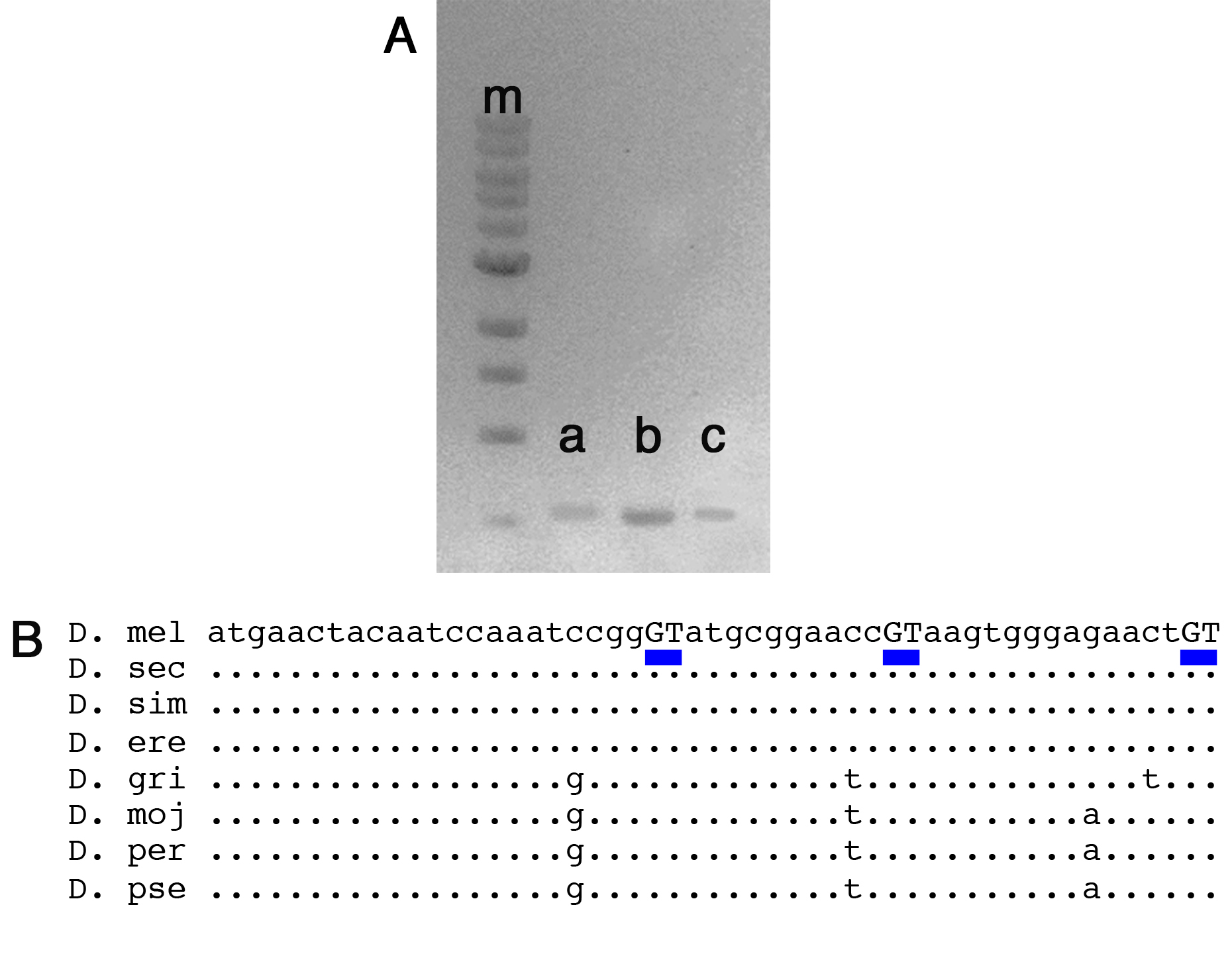

Supplement: Supplemental Information 3 — (A) Agarose gel of reverse-transcriptase PCR amplifications, using isoform-specific primers for splice-types A, B and C described in the CG5484 entry in Flybase. (B) Multiple sequence alignment of genomic DNA at the 3′ end of the first doc exon. Bases identical to D. melanogaster in the other species are indicated with a period; differences are shown by a lowercase letter. The highly conserved GT at the 5′ splice site of intron one necessary to produce the three isoforms in D. melanogaster is capitalized and underlined with blue rectangles; each site is conserved in all seven species of Drosophila shown here, D.mel = D.melanogaster, D. ere = D. erecta, D. gri = D. grimshawi, D. moj = D. mojavensis, D .per = D.persimilis, D. pse = D. pseudoobscura, D. sec = D. sechellia, D. sim = D. simulans. [file peerj-09-12175-s003.jpg]
