# Supplementary material for: A docked mutation phenocopies dumpy oblique alleles via altered vesicle trafficking
Source: PeerJ. 2021 Oct 13;9:e12175. doi: 10.7717/peerj.12175 (PMC8520396; doi:10.7717/peerj.12175)
Supplement: Supplemental Information 4 — Sequence obtained from sequencing heterozygous doc16/doc+ mRNA. The single base change that causes a premature stop codon is highlighted in red. The yellow highlight indicates the region represented by the chromatogram in Fig. 2. [file peerj-09-12175-s004.docx]

Doc16 cDNA Length: 1467

1 AACTCCGGTC ACACAACAAA ATGCTTATGG TGATGTGTTA AAAAGCGAAA

51 TTTTGAATAC AATTTACTGA TCCAGAAAGA TGAACTACAA TCCAAATCCG

101 GCCTCAGCTG CCGGTCGCCC AAGGCCGCCG AAGCGTGTGA GTGATGTTAA

151 CGCCATGGGA CCCTCGGCTC CGATGATGGG CGGTGGCGCC ACCTTCATGG

201 CCCCGCCCAC CGGCCCCGGA ATACTAGATC CCAATATGTA CGGAGCACCT

251 GCTCCGGCCC CGGTCAACAG CTATGGCTTC GACCCCAATC TCGGCCAGCC

301 CTCGCAGCAC ATTCAGCAGC CACCACAACA GCAGCCCGGA TACGGATATG

351 GAGCACCACC TCCCCAACAG GCGGCTGGGC CACCCACCTA CGGAATAGGA

401 GCACCTCAGC CTGTAGCTCC ACCCACTGGA CAGTACCCAC AGTTCGCCAT

451 GTTCCAGCAG CCCATCGTGC AAGACATGGC TATGCAGTAC GGTCAGAAAC

501 TAGCGGATCA GGGCAAGCAG ATCATGGAGA ATTAGTTCGA GAAGTGGGTG

551 CCCGTAGCCA AGCTCAAGTA CTACTTCGCA GTGGACAACG CCTACGTGGG

601 CAGGAAGTTG CGCCTTCTGT TTTTCCCCTA TATGCATAAG GATTGGTCCC

651 TGCGCTACGA CCAAGAGCAC CCAGTGCAAC CACGATATGA TGTTAATGCG

701 CCAGATCTCT ATCTGCCCAC CATGGGCTAC ATCACATACG TGATCGTAGC

751 GGGTTTGCTT CTGGGCATGC AGAAACGATT TTCGCCGGAG CAGCTCGGCA

801 TTCAGGCCTC CAGCGCCATG GCATATAGCA TTTTCGAATT GGTCATTTAC

851 TCCCTAGCCC TGTACGTAAT GAATGTGAAG ACGAGCCTGA AAACGCTTGA

901 TCTGCTGGCA TTCACTGGTT ATAAGTACGT TAATATAGTT GTCTGCCTGA

951 TGGTTAGCAC GCTGTTCTTT AAGTCTGGAT ATTATATAGC ACTGGCGTAT

1001 ACCAGTTTCT CCTTCGGTTT CTTCATGCTG CGCACGTTGC GGACGAAACT

1051 GCTGCAGGAC AACTCCCCGG CTGCGCCCAG TGGAGCCATC AACTACGATC

1101 CGTATGGCAA TCCACAGCAA TTTGACTATA GCGGCGGGAA AAAGCGCAAA

1151 CTCTACTTCC TGTTCATGAT CGTCGCGGGA CAGGCGCTGT TTGCCTTCCT

1201 GCTCTCCAAG CACCTGTATC TGCCCGAGGC GGAGGTGCTG ACGGTGCCCA

1251 AGACCTTCTA ACCCACAGGC TTGATAGGTT AATTTGCATA CTCCTTGAGT

1301 TAATCGTGGC CAGGGTGGCC ACTCCCATGT CGAACGATTC CACAGAGCCT

1351 AGGCATTGTG TTTTCCAGCA TGAAGTTTGT GGTTGTCTCA TTTGAGACTC

1401 GAGCGAGAGA AGCATATTTT TGTTAGAAAC AAACACATTT TATAAATAAA

1451 TACCAGCTAT GAAAACT
